# The Extracellular Vesicles Containing Inorganic Polyphosphate of *Candida* Yeast upon Growth on Hexadecane

**DOI:** 10.3390/jox13040034

**Published:** 2023-09-23

**Authors:** Anton N. Zvonarev, Ludmila V. Trilisenko, Vasilina V. Farofonova, Ekaterina V. Kulakovskaya, Tatiana N. Abashina, Vladimir V. Dmitriev, Tatiana Kulakovskaya

**Affiliations:** 1 Federal Research Center “Pushchino Scientific Center for Biological Research of the Russian Academy of Sciences”, Skryabin Institute of Biochemistry and Physiology of Microorganisms, 142290 Pushchino, Russia; zvonarevibpm@gmail.com (A.N.Z.); luvatri@rambler.ru (L.V.T.); ekaterina.kulakovskaya@gmail.com (E.V.K.); dmitrievibpm@gmail.com (V.V.D.); alla@ibpm.pushchino.ru (T.K.); 2 Federal Research Center “Pushchino Scientific Center for Biological Research of the Russian Academy of Sciences”, Institute for Biological Instrumentation of the Russian Academy of Sciences, 142290 Pushchino, Russia; crazytide@yandex.ru

**Keywords:** hexadecane, *Candida*, polyphosphate, canal, cell wall, extracellular vesicles, DAPI

## Abstract

The cell wall of *Candida* yeast grown on presence of hexadecane as a sole carbon source undergoes structural and functional changes including the formation of specific supramolecular complexes—canals. The canals contain specific polysaccharides and enzymes that provide primary oxidization of alkanes. In addition, inorganic polyphosphate (polyP) was identified in *Candida maltosa* canals. The aim of the work was a comparative study of the features of cell walls and extracellular structures in yeast *C. maltosa*, *C. albicans* and *C. tropicalis* with special attention to inorganic polyphosphates as possible part of these structures when grown on the widely used xenobiotic hexadecane (diesel fuel). Fluorescence microscopy with DAPI has shown an unusual localization of polyP on the cell surface and in the exovesicles in the three yeast species, when growing on hexadecane. Electron-scanning microscopy showed that the exovesicles were associated with the cell wall and also presented in the external environment probably as biofilm components. Treatment of hexadecane-grown cells with purified Ppx1 polyphosphatase led to the release of phosphate into the incubation medium and the disappearance of polyP in vesicles and cell wall observed using microscopic methods. The results indicate the important role of polyP in the formation of extracellular structures in the *Candida* yeast when consuming hexadecane and are important for the design of xenobiotic destructors based on yeast or mixed cultures.

## 1. Introduction

Studying the microorganisms capable of degradation and consumption of petroleum hydrocarbons is one of the most important directions for the creation of new biotechnologies for bioremediation of soils, wastes and natural reservoirs exposed to oil pollution. The *Candida* yeasts [1,2,3] and *Yarrowia lipolytica* [4] are the most studied eukaryotic microorganisms capable of degradation of hydrocarbons. The metabolic pathways of hydrocarbons and their derivatives assimilation by nonconventional yeasts still remain in researchers’ attention [4,5,6,7]. Multiple genes participate in the assimilation of n-alkanes in yeasts [8]. Although the details of metabolic pathways have their own characteristics in yeast of different species, researchers agree that the initial stage of alkane metabolism is their oxidation, carried out by specific cytochromes. The expression of genes belonging to the cytochrome P450 family was increased in *Candida maltosa* grown in the medium with hydrocarbons as sole carbon source [2,9]. It was believed that the alkane-induced cytochrome P450alk, which belongs to the CYP52 family, was responsible for the oxidation of n-alkanes to fatty acids [3,10,11,12]. In *C. maltosa*, eight genes (ALK1-ALK8) belonging to the above protein family were overexpressed when cultivated on n-alkanes as a carbon source [3,13,14]. The yeast *Y. lipolytica* has 12 genes encoding cytochromes of the CYP52 family. Some of them hydroxylate n-alkanes, and the others hydroxylate an ω-terminal end of dodecanoic acid [15]. However, the localization of enzymes catalyzing the primary oxidation of n-alkanes in yeast cells needs further study and clarification. It has been suggested that n-alkanes were transported across plasma membrane of yeast cell and their primary oxidation take place in intracellular compartments, because the cytochrome P 450 was found in the microsomal fraction [16,17] quite a long time ago. The proliferation of the endoplasmic reticulum and an increase in the level of cytochrome P 450alk has been observed in alkane-grown yeast for long time ago [17,18]. For *Y. lipolytica*, alkanes are hydroxylated to the corresponding fatty alcohols and then the alcohols are oxidized to fatty acids in microsomes, mitochondria and peroxisomes, respectively. Degradation of fatty acids to acetyl-CoA via the β-oxidation pathways occurred in peroxisomes, acetyl-CoA, produced in peroxisomes, is converted to C4-compounds by the cooperative action of peroxisomes and mitochondria [3,4]. The lipid transfer proteins are involved in the utilization of n-alkanes and the regulation of cell morphology in response to n-alkanes in *Y. lipolytica* [4].

The question arises how hydrocarbons or their derivatives enter the cell since the role of peroxisomes in the consumption of hydrocarbons has been proven [3,4]. Recently, the transmembrane transport of n-hexadecane by *Candida tropicalis* was revealed [19]. Yeast cells were cultivated with n-hexadecane, and the content of intracellular n-hexadecane and transport dynamics were assayed [19]. The intracellular concentration of n-hexadecane has increased within 15 min, and uptake was inhibited by NaN_3_, an ATPase inhibitor. The uptake kinetics of n-hexadecane corresponds to the Michaelis–Menten model indicating that n-hexadecane transport in the yeast cells is energy-dependent and carried out by a specific transport system [19]. The differentially expressed membrane proteins induced by n-hexadecane are identified and quantified by tandem mass tag labeling coupled with liquid chromatography tandem mass spectrometry analysis. The proteome analysis results demonstrated that proteins responsible for energy production and conversion were among differentially expressed proteins. Protein functional analysis also has suggested that differentially expressed proteins associated with transmembrane transport processes are clearly enriched in endocytosis and phagosome pathways, and the analysis has supported the notion that the underlying transmembrane transport mechanism might be associated with endocytosis and phagosome pathways [19].

Our research develops the concept of a significant role of the cell wall in the processes of consumption of alkanes by yeast cells. It has been shown that a number of ascomycete yeasts, when growing on petroleum hydrocarbons, form modified zones in the cell wall—canals in which oxidative enzymes and cytochrome P-450 are localized, participating in the primary oxidation of these substrates. In addition, it has been found that similar structures are also formed in the yeast cell wall during carbon starvation. Electron microscopic and biochemical studies revealed for the first time the formation of canals in the cell wall during the growth on hydrocarbons [20,21,22,23]. The unique properties of canals—substrate dependence, hydrophobicity, high concentration of oxidizing enzymes and strong chemical and mechanical connection with other components of the cell wall—indicate that these structures are the key components of metabolic interaction with the environment. The high difference in protein profiles of culture liquids and cell wall extracts of *C. maltosa* grown on glucose and hexadecane are revealed [23]. Mass spectrometry has shown that a protein belonging to the cytochrome C family in the NaCl has been extracted from the whole cells grown on hexadecane [23]. Summing up, we assume that the yeast growth on alkanes is accompanied by significant changes in the protein and carbohydrate profile of the cell wall, and the primary oxidation and further transporting of oxidized derivatives into the cell are concentrated in specific molecular machines of the cell wall, called canals. We have obtained a number of cytochemical and microscopic evidence that these structures of *C. maltosa* contain oxidative enzymes related to cytochromes, as well as specific structural polysaccharides and inorganic polyphosphates, which apparently play the role of structural organization and regulation of activity for cytochromes [20,21,22,23]. This concept does not contradict the idea of the important role of endoplasmic reticulum and peroxisomes in the metabolism of alkanes. It is obvious that the formation of canals requires both energy and proliferation of the endoplasmic reticulum and the membrane traffic of proteins, and these processes importance for the consumption of alkanes has been proven [4,19].

Currently, the hypothesis of the relationship between the ability of yeast species to consume petroleum hydrocarbons and their pathogenicity has become widespread. This hypothesis is based on the fact that in both cases there are profound changes in the cell wall, including the formation of biofilms. The universality of channels is indicated by their structural similarity with modified zones of the cell wall of pathogenic yeast of the genus *Candida* under the invasion in a living organism [24,25]. This structural relationship is also expressed in the hydrophobicity of the cell surface and the formation of a biofilm during the transition to a pathogenic state [26,27]. The pathogenicity of the genus *Candida* is due to certain virulence factors, such as the ability to avoid host protection, adhesion, biofilm formation (on host tissue and on medical devices) and the production of tissue-damaging hydrolytic enzymes such as proteases, phospholipases and hemolysin [28]. The formation of biofilms creates clinical problems of concern, since they increase resistance to antifungal drugs, and the mechanism of resistance of biofilms to them is not fully known. This ability can currently be regarded as a pathogenicity factor: microbes inside biofilms acquire pronounced resistance to immune defense effectors and antimicrobial drugs. Therefore, we assume that canals are structures through which these processes can be implemented.

It is known that the cell wall of pathogenic *Candida* species plays a key role in the fungi–host interaction, biofilm formation and drug resistance [29,30,31,32]. There is evidence of a correlation between the ability to consume oil components and pathogenicity for yeast-like fungi of other systematic groups. A collection of black yeast-like fungi from the CBS Fungal Biodiversity Center (Utrecht, The Netherlands) was analyzed, for the ability to grow on hexadecane, toluene and polychlorinated biphenyl as the sole carbon and energy source. Among them, *Exophiala mesophila* and *Cladophialophora immunda* isolated from a patient with chronic sinusitis and a polluted soil sample, respectively, showed the ability to grow on toluene as the sole carbon and energy source, and the capacity to grow on alkylbenzenes has been demonstrated for clinical isolates [33]. The neurotropic black yeast *Exophiala dermatitidis* which inhabits diverse indoor environments was shown to be able to assimilate toluene and hexadecane [34].

It is important to note another aspect that characterizes changes in extracellular structures formed by microorganisms during pathogenesis. It is well known that the composition of biofilms and capsules in pathogenic bacteria such as *Ps. auruginosa*, *N. meningitides*, and others includes polyP (inorganic polyphosphates, linear polymers of orthophosphoric acid), and mutants in the genes encoding polyphosphate kinases are deprived of capsules, the ability to form biofilms, mobility and, as a result, lose pathogenicity [35].

A detailed review is devoted to the problem of the relationship between phosphoric metabolism and pathogenicity in yeast [36]. Phosphorus-rich structures in *Cryptococcus neoformans* were analyzed by combining fluorescence microscopy, biochemical extraction, scanning electron microscopy, electron probe X-ray microanalysis and 3D reconstruction of high-pressure frozen and freeze-substituted cells by focused ion beam-scanning electron microscopy. Intracellular and surface phosphorus-enriched structures were identified. PolyP was essential for capsule assembly, as demonstrated with mutants with defects in the synthesis of this polymer. The demonstration of intracellular and cell wall-associated polyP in *C. neoformans* may lead to future studies involving their participation in both physiologic and pathogenic processes [37]. The impact of a PHO (Phosphate-responsive signal transduction) pathway activation-defective *C. neoformans* mutant (pho81Δ) and a constitutively activated PHO pathway mutant (pho80Δ) on fungal virulence. Irrespective of phosphate availability, the PHO pathway was derepressed in pho80Δ with all phosphate acquisition pathways upregulated and much of the excess phosphate stored as polyP. Elevated phosphate in pho80Δ coincided with elevated metal ions, metal stress sensitivity and a muted calcineurin response, all of which were ameliorated by phosphate depletion. In the pho81Δ mutant, phosphate (Pi), polyP, ATP and energy metabolism were reduced. Blocking the PHO pathway reduced fungal virulence in mouse infection models to a greater extent, and this is most likely attributable to depleted phosphate stores and ATP and compromised cellular bioenergetics [38].

As for *Candida albicans*, a number of data have also been obtained for this pathogen indicating the involvement of PHO pathway components in the processes of cell wall rearrangements and pathogenesis. For example, the protein CaPhm7 in *Candida albicans* localized in the plasma membrane participates in drug resistance and filamentous growth [39]. In *S. cerevisiae* its ortholog is responsible for polyphosphate accumulation [40].

The *C. albicans* high-affinity phosphate transporter Pho84 is required for normal Target of Rapamycin (TOR) signaling, oxidative stress resistance and virulence of this fungal pathogen. It also contributes to *C. albicans*’ tolerance of two antifungal drug classes, polyenes and echinocandins. Echinocandins inhibit biosynthesis of a major cell wall component, beta-1,3-glucan. Pho84 is not present in humans, and targeting of Pho84 and cell wall-constructing enzymes was proposed to be a new strategy for antifungal therapy [41]

*C. maltosa* yeast, which was the main experimental model in our studies of structural and functional adaptations of the cell wall when growing on hexadecane, are not pathogenic. However, back in 1993 [42], the evolutionary position was clarified of *C. maltosa*, an n-alkane-assimilating yeast, by sequencing the nucleotides of the small-subunit ribosomal RNA gene. Phylogenetic analyses showed the close evolutionary relationships of *C. maltosa* with *C. tropicaiis*, *C. viswanathii*, *C. aibicans*, *C. parapsiiosis* and *C. guiiiiermondii*, forming a subgroup within this genus [42]. Biofilms are also used as a structure-forming matrix for the food industry and sorption of heavy metals in bioremediation [43,44,45,46]. These materials have good stability and biocompatibility [47] and have significant potential for various biomedical and biotechnological applications [48,49]. Immobilization of microorganisms or enzymes in the matrix leads to stabilization of their catalytic activity, providing the possibility of repeated or continuous use as biocatalysts [44,50].

Overall, the data discussed above suggest the possibility of some similarities between the adaptation of yeast cells to hydrocarbon consumption and the process of interaction with host cells, such as changes in cell wall structure, biofilm formation and specific changes in phosphorus metabolism.

In this regard, the aim of the work was a comparative study of the features of cell walls and extracellular structures in yeast *C. maltosa*, *C. albicans* and *C. tropicalis*, with special attention to inorganic polyphosphates as possible part of these structures when grown on the widely used xenobiotic hexadecane (diesel fuel).

## 2. Materials and Methods

### 2.1. Strains and Growth Conditions

Yeast *Candida albicans* Y-2994, *Candida tropicalis* Y-2771, *Candida maltosa* Y1506 were obtained from All-Russian Collection of Microorganisms “https://vkm.ru/index.htm (accessed on 20 September 2023)”. The cells were cultured to the stationary growth stage at t 28 °C with stirring (150 rpm) in a YNB medium (Difco, Sparks, MD, USA) containing 1% glucose or 1% hexadecane as a carbon source and 1.5 g/L yeast extract (Difco, Sparks, MD, USA). The culture reached the stationary growth phase in the case of glucose for 48 h, and in the case of hexadecane for 96 h.

### 2.2. Fluorescent Microscopy

To stain the polyP, the cells were incubated with 0.05 mg/mL DAPI (1,4′,6′-diamino-2-phenylindole 2HCl) (Sigma, St. Loui, MI, USA) at room temperature for 15 min. The cells were examined using phase contrast and fluorescence microscopy in AXIO Imager A1 (Carl Zeiss AG, Oberkochen, Germany) with a set of filters 56HE and a set of filters 49 () at a wavelength of 365 nm (excitation) and 512 + 630 nm (emission). An Axiocam 506 (Carl Zeiss AG, Oberkochen, Germany) camera was used to obtain the images.

### 2.3. Scanning Electron Microscopy

The surface morphology of the cells was examined with scanning electron microscopy (SEM). Samples of the cells placed on membrane filters were fixed at 4 °C for 24 h in glutaraldehyde vapor and post fixed at 20 °C for 3 h in OsO_4_ vapor. After dehydration in propylene oxide vapor, the samples were coated with gold (Fine Coat Ion Sputter JFC-1100, Tokyo, Japan) and examined under a scanning microscope JSM-6510LV (JEOL, Tokyo, Japan).

### 2.4. Phosphate and Polyphosphate Extraction and Assay

The extraction and assay of Pi and acid-soluble and acid-insoluble polyP in the biomass were carried out according to the method described earlier [51]. Biomass samples were separated from the culture medium using centrifugation at 5000× *g* for 15 min and washed twice with distilled water using centrifugation at 5000× *g* for 15 min. Acid-soluble polyP and Pi were extracted from biomass with 0.5 M HClO_4_ at 0 °C as described earlier [51,52]. The Pi in extracts was assayed with a colorimetric method [53]. Then, the extracts were treated with 0.5 M HClO_4_ at 90 °C for 20 min and polyP content was estimated from the amount of additional phosphate released [52]. The amount of acid-insoluble polyP was estimated in biomass samples remained after above treatment. The samples were treated with 0.5 M HClO4 at 100 °C for 20 min [51]. The released phosphate was measured with a colorimetric method [53]. The amount of polyP was calculated as released phosphate amount per g of wet biomass. All biomass samples were obtained with standard centrifugation conditions applied in our previous studies, 5000× *g* for 15 min [51].

### 2.5. The Treatment of Biomass with Polyphosphatase Ppx1

The yeast strains Y-2994, Y-1516 and Y-2771 were cultured in YNB medium containing hexadecane for 96 h. Then samples of 1 mL of culture were taken and the cells were separated using centrifugation at 7000× *g* for 5 min. The cell samples were washed 2 times with 0.5 mL of 50 mM Tris HCl, pH 7.0 and precipitated at the same conditions. The resulting cell sediments were used for treatment with purified polyphosphatase Ppx1. The enzyme preparation was purified as described earlier [54] and stored at −20 °C in 50 mM Tris HCl, pH 7.0. Its activity was 3.46 ± 0.065 U per 1 mL. The incubation medium contained 0.01 mL of 200 mM Tris HCl, pH 7.0, 0.005 mL of 100 mM MgSO_4_, 0.01 mL of 2M NH_4_Cl and 0.05 mL of Ppx1 preparation. In the control samples, 0.05 mL of 50 mM tris HCl, pH 7.0 was added instead of the enzyme preparation. The samples were incubated in a thermostat at 30 °C with stirring for 2 h. After that, cells were precipitated with centrifugation at 7000× *g* for 5 min and used for polyP and Pi extraction and assay. Supernatants were used to measure the amount of released Pi.

Extraction and assay of Pi and acid-soluble and acid-insoluble polyP from biomass samples was carried out according as described in Section 2.4. Pi was assayed using malachite green colorimetric method [54].

### 2.6. Amino Acid Sequences Alignments

The data were obtained through Basic Local Alignment Search Tool for proteins “https://blast.ncbi.nlm.nih.gov/Blast.cgi?PAGE=Proteins (accessed on 20 September 2023)”. Validated AA sequences from the Saccharomyces Genome Database (SGD, strain S288C): *VTC4* (SGD:S000003549), *PPX1* (SGD:S000001244), *PPN1* (SGD:S000002860) and *PPN2* (SGD:S000005161), were used as a reference sequence for searching homologous protein sequences in *Candida* species through a blastp algorithm (protein–protein BLAST) with default settings. Comparative analysis of protein sequences was also performed using a blastp algorithm. All *Candida* proteins accession numbers are valid for NCBI database. *Candida albicans* proteins belong to four different strains (*VTC4*—SC5314 strain, *PPX1*—P37039, *PPN2*—P75016, *PPN1*—P76067), *Candida tropicalis*—strain *Candida tropicalis* MYA-3404 and *Candida maltosa*—*Candida maltosa* Xu316.

### 2.7. Statistical Evaluation

The results are shown as the mean and standard deviation (SD) of at least three separate experiments, and we present the *p*-values generated by this data. The standard Excel program was used for this analysis.

## 3. Results and Discussion

### 3.1. Fluorescent and Scanning Electron Microscopy of Yeasts Cells

**Figure 1:**
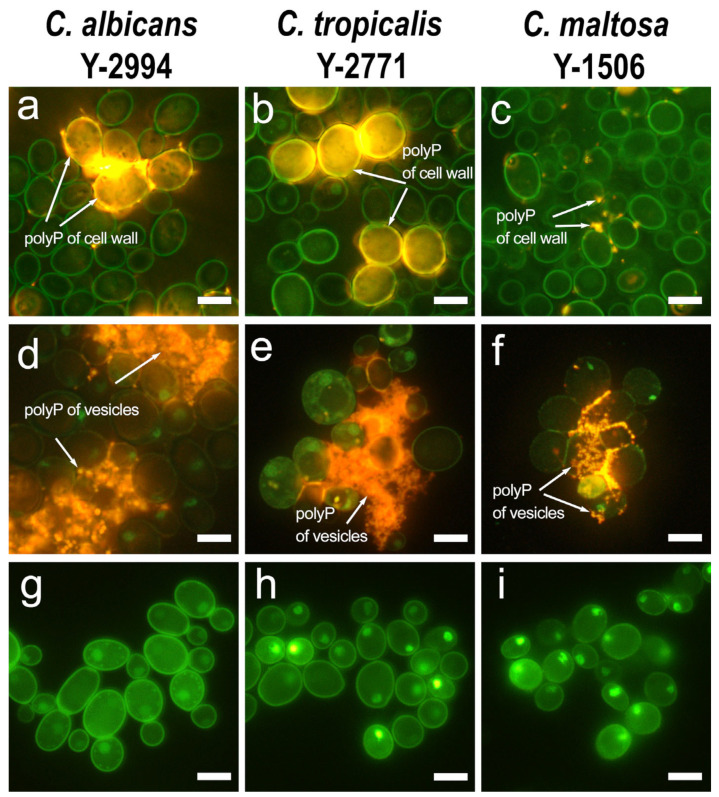
Fluorescent microscopy with DAPI staining for polyphosphates: (**a**) (*C. albicans*), (**b**) (*C. tropicalis*), (**c**) (*C. maltosa*)—cells grown on a medium with glucose; (**d**) (*C. albicans*), (**e**) (*C. tropicalis*), (**f**) (*C. maltosa*) —cells grown on a medium with hexadecane; (**g**) (*C. albicans*), (**h**) (*C. tropicalis*), (**i**) (*C. maltosa*)—treatment with Ppx1 polyphosphatase. Scale bar 5 µm.

DAPI staining is a convenient and specific fluorescence method to detect not only DNA (0.1–1 μg/mL DAPI), but also polyP (3–50 μg/mL DAPI) [55,56]. Long-chained polyP forms with DAPI complex with orange fluorescence, short-chained polyP-DAPI complex has green fluorescence, while DNA–DAPI complex has blue fluorescence [55,56]. Earlier, using DAPI staining and treatment of the cells with polyphosphatase, we obtained evidence of the presence of polyP in the canals of an another *C. maltosa* strain cultivated on hexadecane [22]. Fluorescence microscopy with DAPI showed unusual localization of polyP on the cell wall surface *Candida* yeast under study (Figure 1). In the case of glucose grown cells, a part of the population had an orange fluorescence of the cell walls indicating the presence of long-chained polyP. The cells cultivated in hexadecane containing medium had a discrete arrangement of short-chained polyP in the cell wall, revealed by green fluorescence (Figure 1a–c). However, a large number of spherical exovesicles were also found in the external environment with an orange fluorescence indicating the presence of a long-chained polyP (Figure 1d–f). Treatment of hexadecane-grown cells with yeast polyphosphatase Ppx1, an enzyme highly specific to polyP degradation [57], completely removed the orange fluorescence of cell walls and exovesicles, which confirmed the presence of long-chained polyP in these structures (Figure 1g–i). It is unlikely that polyphosphatase Ppx1, an enzyme with a molecular mass of 40 kD, could penetrate into the cell through the cytoplasmic membrane. Microscopy (Figure 1) shows that after Ppx1 treatment, the cells retain their intactness, while the DAPI-stained compounds of extracellular vesicles and cell wall disappeared. Therefore, Ppx1 in our experimental conditions most likely destroyed polyP both in the vesicles and in the cell wall.

**Figure 2:**
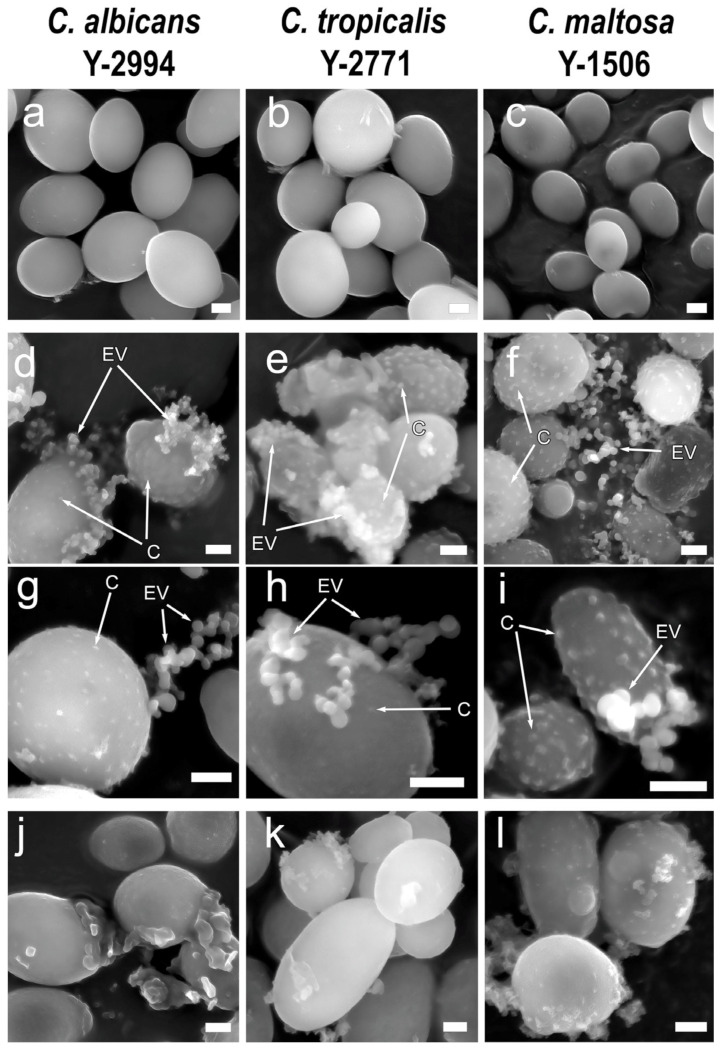
Scanning electron microscopy: (**a**) (*C. albicans*), (**b**) (*C. tropicalis*), (**c**) (*C. maltosa*)—cells grown on a medium with glucose; (**d**) (*C. albicans*), (**e**) (*C. tropicalis*), (**f**) (*C. maltosa*)—cells grown on a medium with hexadecane; (**g**) (*C. albicans*), (**h**) (*C. tropicalis*), (**i**) (***C****. maltosa*)—cells grown on the medium with hexadecane (higher magnification then on (**d**–**f**)); (**j**) (*C. albicans*), (**k**) (*C. tropicalis*), (**l**) (*C. maltosa*)—treatment with Ppx1 polyphosphatase. C—canals of cell wall, EV—extracellular vesicles. Scale bar 1 µm.

Electron microscopic analysis of the cell surface and their environment during growth on glucose revealed as expected nothing unusual (Figure 2a–c). The cells utilizing hexadecane had modified cell wall zones on the surface (canals) similar to observed earlier in hexadecane-grown *C. maltosa* [20,21,22,23]. The presence of extracellular vesicles was also confirmed, as well as with fluorescence microscopy. The exovesicles of all three species had approximately the same size and shape (Figure 2d–f). Treatment of cells with Ppx1 polyphosphatase also showed destruction of canals and exovesicles. At the same time, the detachment of the outer layer of the cell wall was detected (Figure 2g–i). Thus, scanning microscopy in combination with treatment with polyphosphatase confirmed the necessity of the presence of polyP in the canals and extracellular vesicles of three yeast species under study.

### 3.2. Polyphosphate Content in the Cells Cultivated on Glucose or Hexadecane

We determined the content of Pi, acid-soluble polyP and acid-insoluble polyP in *C. maltosa*, *C. albicans* and *C. tropicalis* cells grown on both glucose and hexadecane. We used an extraction method which allows the differentiation of acid-soluble polyP with an average chain length of 15 to 25 phosphate residues and acid-insoluble polyphosphates with an average chain length of 45 to 200 or more phosphate residues [22,52]. There is evidence that acid-insoluble polyPs, which can be obtained in several fractions with yeast cell treatment with saline and alkaline solutions, are associated with subcellular fractions, including vacuoles and cell walls [52]. This was believed to be the reason for their insolubility in acidic solutions [52].

It should be noted that the biomass yield on the two media differed significantly. The yield of wet biomass on glucose for all three species was 22–23 g/L, while on hexadecane this yield was significantly lower and did not exceed 4–6 g/L. As for the content of polyP, it was not possible to find any regularity characteristic of the three studied species. The total content of polyP increased by 53% in *C. maltosa* and by 60% in *C. tropicalis* but decreased by 30% in *C. albicans*. In *C. albicans*, the content of acid-insoluble polyP decreased, but the content of acid-soluble polyP increased. In *C. maltosa* and *C. tropicalis*, the main contribution to the increase in the content of polyP was made by the acid-insoluble fraction (Table 1). An increase in the content of the acid-insoluble fraction was observed earlier for another strain of *C. maltosa* cultivated on hexadecane [23].

### 3.3. The Treatment with Polyphosphatase

**Figure 3:**
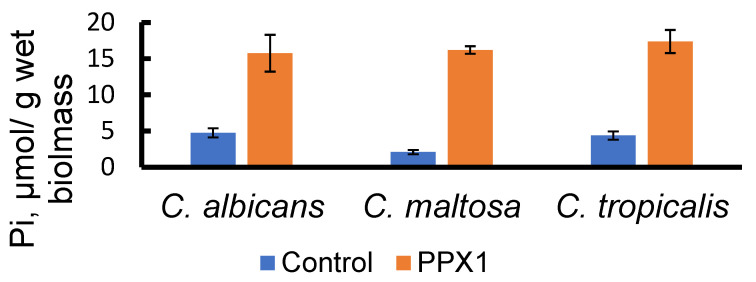
Pi release to the medium after incubation of biomass of three yeast species under control conditions and in the presence of Ppx1 polyphosphatase. The cells were grown in a medium with hexadecane. The experiments were performed in 3 replicates; the values denote mean and the error bars denote s.d. *p* < 10^−4^ in all cases, significance was assessed with the one-tailed Student’s *t*-test performed for Ppx1 treatment versus control incubation of biomass samples.

**Figure 4:**
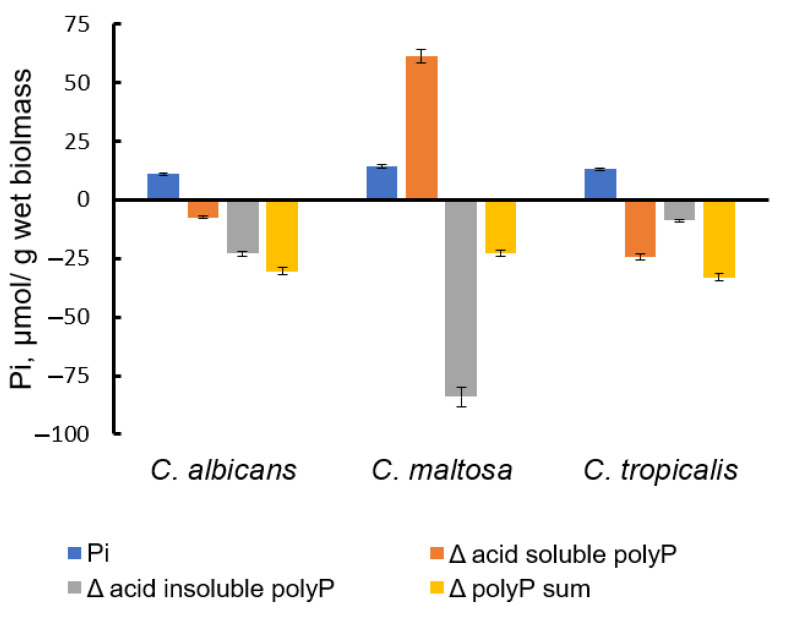
The amount of Pi released into the medium from the biomass, and changes in the amount of acid-soluble polyP and acid-insoluble polyP in biomass of three yeast species after incubation in the presence of Ppx1 polyphosphatase. The cells were cultivated in the medium with hexadecane. The experiments were performed in 3 replicates; the values denote mean and the error bars denote s.d. *p* < 10^−4^ in all cases, significance was assessed with the one-tailed Student’s *t*-test performed for Ppx1 treatment versus control incubation of biomass samples.

In order to assess the content of extracellular polyP in the biomass, we applied the treatment of biomass samples with a polyphosphatase Ppx1 from *S. cerevisiae*. This enzyme does not hydrolyze nucleoside phosphates and pyrophosphate, but it is capable of cleaving polyP with different chain lengths from three to hundreds of phosphate residues [54]. Note, that according to microscopy data (Figure 1), the cells remain intact under this treatment, and the likelihood of penetration of a 40 kDa Ppx1 protein through the cell wall is unlikely. Incubation of biomass samples at neutral pH and 30 °C resulted in a small release of phosphate into the medium. When treated under the same conditions with Ppx1, the release of phosphate increased almost fourfold (Figure 3). At the same time, in the biomass of all three species, the total content of polyP was lower in the samples treated with Ppx1 compared to the control ones (Figure 4).

In all three species, the reduction affected the fraction of acid-insoluble polyP. This suggests that the extracellular polyP is protected from acid extraction either by lipids and/proteins of vesicles or by binding to proteins and polysaccharides in the cell wall. At the same time, this polyP was available for enzymatic hydrolysis by Ppx1.

## 4. Discussion

This work, using fluorescence and microscopy methods in combination with the enzymatic assay of polyP, shows that the cells three *Candida* species cultured in a medium with hexadecane form extracellular vesicles containing PolyP. Apparently, such structures are involved in the formation of biofilms necessary for interaction with such insoluble hydrophobic substrate as hexadecane which presents in the medium as microdrops of the hydrophobic phase. In addition, polyP seems to be part of canals, a specific structure of the cell wall. In this study we revealed such structures not only in *C. maltosa*, but also in other *Candida* species.

The origin of the extracellular polyP is of special interest, since no similar structures have been found in the yeast *S. cerevisiae*, where the polyP metabolism and cellular localization has been studied to the greatest extent. According to modern concepts, polyP in yeast is synthesized by the Vtc4 polyphosphate synthase, which is part of the VTC (vacuolar transporter chaperone) complex [58,59]. There are two forms of VTC complex present in the yeast cells: Vtc4/Vtc3/Vtc1 and Vtc4/Vtc2/Vtc1 [60]. The former is typical of the vacuolar membrane and the latter is typical of the endoplasmic reticulum membrane and nuclear envelope [60]. This complex synthesizes long-chain polyP [58], which can then split into short-chain through hydrolysis by endopolyphosphatases Ppn1 and Ppn2 [53]. Apparently, this complex in the membrane of the endoplasmic reticulum should be responsible for the synthesis of polymers localized in the cell wall and extracellular structures. In this case, the traffic of these polymers into the cell wall and extracellular space should be carried out by means of transport vesicles of various types.

We compared the sequences of *VTC4*, *PPN1* and *PPN2* genes in three studied species using the sequences of *S. cerevisiae* as a comparative sample (Table 2). Vtc4 sequences have significant similarities, while the sequences of three polyphosphatases, both exopolyphosphatase PPX1 and both endopolyphosphatases show a lower level of similarity with the sequences of *S. cerevisiae*. The similarity of above sequences between *Candida* species used in this study was more pronounced (Table 3). Currently, there are no data in the literature on the properties and features of the regulation of enzymes of polyP metabolism in *Candida* yeasts. Therefore, we have no convincing hypothesis on the structural features of these proteins associated with the ability of *Candida* cells to form a significant pool of extracellular polyPs. The analysis of such features is of interest for future research in order to understand the interaction of these yeasts with the environment. It is possible that the observed differences may be related to differences in the regulatory regions of these enzymes, which causes a decrease in the polyP chains fragmentation and increase in content of extracellular long-chain polyP when growing on hexadecane.

Extracellular vesicles (EVs) are membranous, rounded vesicles formed by prokaryotic and eukaryotic cells both at normal growth and at growth in unfavorable environments [61]. These vesicles are a part of a system of communication between cells and are involved in cell wall formation and functioning, supporting cell integrity, isolation and resistance against the environment. In the case of pathogenic strains, they might take part in the interactions with the host and affect the infection outcomes [61]. In *Candida albicans* and *Candida haemulonii,* fungal extracellular vesicles (EVs) play a critical role in the pathogenicity and virulence and perform specific functions during infections, such as host cells interaction, transport of virulence factors and influence on cell survival in host organism [62,63].

Under interaction of *Candida* cells and mammalian cells, multiple changes in gene expression and in cellular morphology appeared, for example the expression of virulence factors (adhesins, invasins and extracellular hydrolytic enzymes), the transition to filamentous hyphae form and the formation of biofilm [64,65,66]. Researchers are increasingly interested in the role of PHO regulon [67] and the extracellular matrix [68] in these processes.

The study of the role of polyP in the organization of extracellular structures in yeast is of importance in the search for new mechanisms of adaptation to adverse conditions. We studied extracellular polyphosphates during the growth of three yeast species on hexadecane. Considering that *C. maltosa* is not a pathogenic strain, we assume that the observed effects in our case are associated with the need to consume a hydrophobic substrate, hexadecane. We have no reason to assert that the observed effects are related to pathogenicity. However, the formation of extracellular vesicles and biofilms is characteristic of pathogenic yeast, and we believe that in further studies in other experimental models, including models of interaction of yeast cells with mammalian cells, it is of interest to pay special attention to the presence of inorganic polyphosphates in the cell wall and extracellular structures, including biofilms.

## 5. Conclusions

This study shows that *Candida* yeast, when cultured on hexadecane, forms not only special cell wall structures but also extracellular vesicles containing inorganic polyphosphates. These polyphosphates are stained with DAPI and are available for enzymatic hydrolysis by phosphatase. Microscopic studies have shown that treatment with polyphosphatase leads to the destruction of these structures, which indicates the important role of polyP in their formation and functioning. The data obtained in this work that long-chain polyP were included in the extracellular vesicles under hexadecane consumption indicate these polymers as a component of vesicles and biofilms formed by yeasts. Such extracellular structures participate in pathogenic processes caused by yeast, and the future studies of polyP role in fungal pathogenicity may open new ways in the development of new drugs against fungi pathogens. For example, yeast polyphosphate synthetase, responsible for polyP biosynthesis, is absent in humans and may be of promising interest as a target of new drugs against candidiasis. The use of hexadecane as a model xenobiotic expands the understanding of its destruction by microorganisms in the natural environment and can also help in future medical research.

## Figures and Tables

**Table 1:** The content of Pi, acid-soluble and acid insoluble polyP in yeast cells grown on glucose or hexadecane, μmol P/g wet biomass.

	Glucose			Hexadecane		
Yeast Species	Pi	Acid Soluble polyP	Acid Insoluble polyP	Pi	Acid Soluble polyP	Acid Insoluble polyP
*C albicans*	10.6 ± 0.20	22.0 ± 1.93	115 ± 4.1	27.6 ± 1.625	34.0 ± 1.26	59.1 ± 13.4
*C. tropicalis*	10.3 ± 0.25	3.93 ± 0.66	30.5 ± 1.0	14.9 ± 0.23	10.9 ± 0.91	44.8 ± 4.97
*C. maltosa*	15.8 ± 2.4	8.02 ± 2.04	79.6 ± 1.1	16.3 ± 1.2	11.5 ± 4.24	124 ± 9.9

**Table 2:** The AA sequence alignment of *VTC4*, *PPX1*, *PPN1* and *PPN2* for *C. albicans*, *C. tropicalis* and *C. maltosa* by BLAST with sequences of *S. cerevisiae* as a reference.

	Species	*C. albicans*	*C. tropicalis*	*C. maltosa*
*VTC4* (SGD:S000003549)	Accession number	XP_711583.1	XP_002545726.1	EMG48228.1
	Query Cover, %	99	99	99
	Percent identity	60.82	60.27	60.95
*PPX1* (SGD:S000001244)	Accession number	KHC58625.1	XP_002547193.1	EMG50694.1
	Query Cover, %	97	90	95
	Percent identity	32.78	34.36	34.2
*PPN2* (SGD:S000005161)	Accession number	KHC62723.1	XP_002550886.1	EMG47562.1
	Query Cover, %	95	95	96
	Percent identity	29.01	30	29.14
*PPN1* (SGD:S000002860)	Accession number	KHC31142.1	XP_002550589.1	EMG47375.1
	Query Cover, %	94	93	87
	Percent identity	41.01	40.59	42.95

**Table 3:** The AA sequence alignment of *VTC4*, *PPX1*, *PPN1* and *PPN2* for *C. tropicalis* and *C. maltosa* by BLAST with sequences of *C. albicans* as a reference.

	Species	*C. tropicalis*	*C. maltosa*
*VTC4* (XP_711583.1)	Accession number	XP_002545726.1	EMG48228.1
	Query Cover, %	99	99
	Percent identity	82.71	86.85
*PPX1* (KHC58625.1)	Accession number	XP_002547193.1	EMG50694.1
	Query Cover, %	100	100
	Percent identity	61.94	63.03
*PPN2* (KHC62723.1)	Accession number	XP_002550886.1	EMG47562.1
	Query Cover, %	100	100
	Percent identity	59.95	59.44
*PPN1* (KHC31142.1)	Accession number	XP_002550589.1	EMG47375.1
	Query Cover, %	97	99
	Percent identity	65.1	64.05

## Data Availability

Not applicable.
